# Editorial: Women in Analytical Chemistry

**DOI:** 10.3389/fchem.2022.949793

**Published:** 2022-08-11

**Authors:** N. J. Jaffrezic-Renault, O. Giuffrè, E. Gallardo, C. Bala, Q. B. Cass

**Affiliations:** ^1^ Université Claude Bernard Lyon 1, Villeurbanne, France; ^2^ Dipartimento di Scienze Chimiche, Biologiche, Farmaceutiche ed Ambientali, Università di Messina, Messina, Italy; ^3^ Centro de Investigação em Ciências da Saúde (CICS-UBI), Faculdade de Ciências da Saúde, Universidade da Beira Interior, Covilhã, Portugal; ^4^ Department of Analytical Chemistry, University of Bucharest, Bucharest, Romania; ^5^ Departamento de Química, Universidade Federal de São Carlos, São Carlos, Brazil

**Keywords:** analytical chemistry, clinical, food, agriculture, speciation, sensors

This collection encompasses different areas of analytical chemistry and highlights the contribution and research work of women scientists from different parts of the world. The collection is composed of 18 articles and involves clinical applications, food and agricultural products analysis, speciation studies and analysis, semiconductors, and novel sensors developments.

## Clinical applications

The *in vitro* method for the prediction of drug bioavailability is of importance since it can save time, money, reduce chemical and biological waste, and when well-designed can be used for planning *in vivo* bioequivalence evaluation (BE). A dissolution/permeation model has herein been reported for 1.5 mg of levonorgestrel (LVN) as generic and brand-name tablets (Simone et al.). To meet this end, a standard dissolution test was combined with a parallel artificial membrane permeability assay (PAMPA). From this work, PAMPA has emerged as a complementary assay for predicting *in vivo* BE of drugs and explained the unsuccessful *in vivo* bioequivalence fallouts for these two tablets.

The ability to quantify amphotericin B (AmB) at any given time during the treatment of neurocryptococcosis is an analytical challenge, especially in cerebrospinal fluid (CSF). Lanchote and collaborators report on the development of a LC-MS/MS method for analysis of AmB in plasma, plasma ultrafiltrate, urine, and CSF (Francisco Pippa et al.). The developed method has been applied to a clinical pharmacokinetic study following the administration of the drug as a lipid complex in one patient. With that, a detailed description of the pharmacokinetic parameters has been obtained and created the conditions to investigate AmB in CSF at any time of the treatment.

The increased number of antibodies and derivatives on the market calls for methods development for analyzing biosimilars. Herein, capillary gel electrophoresis (CGE), liquid chromatography on the reversed elution mode (RPLC), and size-exclusion chromatography (SEC), all with UV detection, were used to analyze the monoclonal antibody (adalimumab), an Fc-fusion protein (etanercept) and to compare them to one of their biosimilars (Demelenne et al.). The three separation methods showed analytical complementarity for the biopharmaceuticals. While RPLC was useful for the separation of hydrophilic and hydrophobic degradation products, CGE showed selectivity for several adalimumab fragments, and SEC was worthwhile for the analysis of aggregates and certain fragments. LC-HRMS was also used to determine the exact mass of the intact protein of adalimumab while SEC with multiangle light scattering detection was used to measure the MW of etanercept (150 kDa) without overestimation. The article meets the requirement of finding innovative analytical conditions especially for Fc-fusion proteins.

The GABAB receptor is a typical G protein-coupled receptor, and its functional impairment is related to a variety of diseases such as anxiety, depression, alcohol addiction, Parkinson, and cancer. While the premise of GABAB receptor activation is the formation of heterodimers, the receptor also forms a tetramer on the cell membrane. Thus, it is important to study the effect of the GABAB receptor aggregation state on its activation and signaling. In this study, Luo et al. have applied single-molecule photobleaching step counting and single-molecule tracking methods to investigate the formation and change of GABAB dimers and tetramers. A single-molecule stoichiometry assay of the wild-type and mutant receptors revealed the key sites on the interface of ligand-binding domains of the receptor for its dimerization. Moreover, they found that the receptor showed different aggregation behaviors under different conditions. The results offered new evidence for a better understanding of the molecular basis for GABAB receptor aggregation and activation.

Cardiovascular diseases have complex etiology and high mortality rates. Their main risk factors increase the formation of reactive oxygen species, normally produced by natural processes of cellular metabolism. Amaral et al. aim to perform an untargeted metabolomics investigation of cardiac cells (H9c2). The cells were divided into three groups: healthy cells, cells after simulated oxidative stress by H_2_O_2_, and cells after recovery from oxidative stress. The purpose of this work was to identify changes in metabolism between groups. After some *in vitro* assays, the authors used liquid chromatography coupled with mass spectrometry (RPLC-MS and HILIC-MS) analyses to know metabolite annotation and pathway analysis. This multiplatform analysis in untargeted metabolomics approaches showed its importance in this field.

Enzymes immobilized on solid supports have received increasing attention as tools in clinical diagnosis because of their advantages like improved stability and the possibility of reusing proteins. Such screening assays are based on bioaffinity chromatography, which combines the specificity and sensitivity of an enzymatic reaction with the automation and reproducibility of a chromatographic system. Current approaches for high-throughput screening (HTS) are based on the search for compounds with efficacy toward single targets. However, a new paradigm is emerging in pharmacological research-exploration on the bioactivity of compounds at multiple targets. Lopes Vilela et al. developed an innovative, automated dual enzymatic system assay based on acetylcholinesterase and beta-secretase 1 co-immobilized on the inner surface of a fused silica capillary to screen ligands. To measure enzyme activities, liquid chromatography coupled to ion trap mass spectrometry was used. The proposed method was validated in order to confirm the standard inhibitors for both enzymes by determining half-maximum inhibitory concentrations. This assay proved to be an excellent tool for multitarget-directed ligands in drug discovery for complex diseases, and an improvement on the HTS technique as well.

Concerning clinical imaging for diagnosis, staging, and therapy planning, there has been a great deal of attention concerning indocyanine green (ICG) dye. ICG is one of the most commonly used fluorophores in near-infrared fluorescence-guided techniques. However, the molecule forms aggregates in saline solution, presenting limited photostability and moderate fluorescence yield. Sottani et al. formulated ICG using protein-based nanoparticles of H-ferritin (HFn) to generate a more stable fluorophore (HFn-ICG). Ultrahigh performance liquid chromatography-tandem mass spectrometry was employed to determine ICG in liver samples from HFn-ICG-treated mice. The method was validated and applied successfully in bio-distribution studies to investigate the delivery of HFn-ICG.

*Pseudomonas aeruginosa* is a Gram-negative multidrug-resistant pathogen causing acute and chronic infections. L. Vilaplana *et al.* report on the development of a reliable, highly sensitive, and specific, immunochemical assay to detect pyocyanin (PYO), one of the main virulence factors (VFs) of *Pseudomonas aeruginosa* (Rodriguez-Urretavizcaya et al.). The ELISA developed allows researchers to achieve a limit of detection, LoD, in the low nM range. The strengths of this study are the potential of PYO as a biomarker of *P. aeruginosa* infection in clinical samples and the possibility of assessing the nature of the infection stage of the patients.

## Food and agricultural products analysis

The chromatographic profile of the ethanolic extract of *Bauhinia forficata* leaves was developed as a model through a design of experiment (DoE) by modifying simultaneously multiple critical method parameters to find the appropriated chromatographic conditions (Aquino et al.). Then, they were used by LC-HRMS to identify the compounds that could differentiate the ethanolic extracts leaves of four Bauhinia Species: *B. forficata*, *B. variegata*, *B. longifolia*, and *B. affinis.* The MS data of the chemical profile evaluated by principal component and hierarchical cluster analysis was able to differentiate the species and, thus, be used for authentication of these herbal medicines. Furthermore, 55 molecules were inferred by dereplication. For that, manual comparison of the exact masses, MS/MS fragmentation patterns, and isotopic contribution patterns with those data either reported in the literature or deposited in spectral libraries online were carried out. The used analytical protocol has met the principles of green chemistry and has contributed to the phytochemical knowledge of the studied *Bauhinia* species and can be used for other natural product libraries.

Đurđić et al. analyzed different samples of Serbian wine in order to detect lead isotope ratio patterns. The authors proposed that the lead isotope ratio is an excellent “*fingerprint*” to access information on the geographical origin of wine as well as to identify potential sources of lead pollution. Through inductively coupled plasma mass spectrometry and multivariate methods of analysis, the authors were capable of comparing the levels of lead in Serbian wines with those of wines from different origins. Several anthropogenic sources are also shown to contribute to the total isotopic profile of lead. Taking into account the results obtained, it was possible to determine the authenticity and geographical origin of the wine using isotopic lead profiles.

Pérez-González et al. developed a portable potentiometric tongue (PE-tongue) and applied it to evaluate the quality of milk with different fat contents (skimmed, semi-skimmed, and whole) and with different nutritional content (classic, calcium-enriched, lactose-free, folic acid-enriched, and enriched in sterols of vegetal origin). The system consisted of a simplified array of five sensors based on PVC membranes, coupled to a data logger. Principal component analysis (PCA) and support vector machine (SVM) results indicated that the PE-tongue consisting of a five-electrode array could successfully discriminate and classify milk samples according to their nutritional content. SVM regression models were used to predict the physicochemical parameters classically used in milk quality control (acidity, density, % proteins, % lactose, and % fat). The prediction results were excellent and similar to those obtained with a much more complex array consisting of 20 sensors.

Finally, Rocha et al. performed a review in order to discuss the potential of the comprehensive two-dimensional gas chromatography methodologies, combined with a headspace solvent-free microextraction technique, in tandem with data processing and data analysis as a useful tool to the coverage of chemical aroma clouds of foods. Also due to the chemical complexity of aromas, the authors presented some challenges related to the characterization of volatile molecules and the perception of aromas as well as some examples reported in recent publications.

## Speciation studies and analysis

The determination of Hg size speciation and the amount of Hg bound to dissolved organic matter (DOM) is crucial to assessing its fate and bioavailability in natural surface waters. Worms *et al.* describe a methodology combining thiol labeling by fluorescent monobromo(trimethylammonio)bimane bromide (qBBr) with asymmetrical flow field-flow fractionation and online fluorescence detection (AF4-FluoD), for separation of components, determination of size distribution and content of this in the macromolecular DOM in natural waters (Worms et al.). After enrichment with mercury, the quantification and the characterization of the size distributions of Hg bound to macromolecules and nano-sized inorganic particles were made using AF4-ICP-MS. The results of this study highlight new opportunities for determining the stability of the Hg-DOM complex.

The performance of biomaterials, such as O-phosphorylcholine (PPC) based materials, employed for clinical applications, can be affected by electrolytes. Giuffrè *et al.* describe a multidisciplinary study useful to elucidate the interaction between O-phosphorylethanolamine (PEA) and PPC, compounds constituting the headgroups of biomembranes, with Mg^2+^ (Aiello et al.). The acid-base behavior, the complexation properties, the enthalpy changes, and the speciation were obtained by potentiometry and ^1^H-NMR spectroscopy. Spectra obtained by matrix-assisted laser desorption mass spectrometry (MALDI-MS) and MS/MS on these systems indicated an interaction mechanism *via* the phosphate group giving rise to a four-membered cycle. The purpose of the study was mainly to obtain thermodynamic information necessary to carry out simulations under real biological fluid conditions, and for evaluating the possible use of these compounds in several application fields.

## Semiconductors

In the field of semiconductors, gallium sulfide (GaS) is part of the new two-dimensional (2D) materials of significant interest for optoelectronic applications. A systematic study on the dependence of several properties on the thickness of layered GaS has been performed by Gutiérrez et al.. The GaS properties are structural from Raman spectra, photoluminescence, optical transmittance, resistivity, and work function (WF). More in detail, Raman spectra measured in layers show that the intensity increases with thickness. The resistivity decreases under visible and UV illumination showing a significant dependence on GaS thickness. The analysis of the WF shows an increase from Bulk GaS to monolayer. The results of this investigation may be useful for designing GaS-based optoelectronic devices.

## Novel sensors

Electrochemical sensors and biosensors play a significant role in delivering data direct, in complex samples without prior separation of the target analyte, with applications in various fields ranging from the environment and food monitoring, clinical laboratory, and industrial applications. Shalini Prasad *et al.* (Narayanan Dhamu et al.) report a rapid response (10 min) sensors design and setup to screen two commonly used pesticides, glyphosate and atrazine, in soil. The reported sensor functions on the bio-affinity mechanism driven *via* an antibody receptor, specific towards the target pesticide. This development proves to be robust for a point-of-use (PoU) setting yielding LoD levels of 0.001 ng/ml for atrazine and 1 ng/ml for glyphosate.

The presence of antibiotic residues in various environmental samples, including water samples, represent an ecological risk. Herein, a notable contribution was done by Priscilla Baker’s research group (Hamnca et al.) which reports a sensor modified with polyamic acid nanofibers to detect sulfonamides, classified as bacteriostatic antibiotics which can act as contaminants in the aquatic environment, for which there are no regulatory guidelines. The reported sensors succeed to perform real-time detection of sulfonamides within a wider linear range and lower applied potential.

Water detection in organic solvents is of great interest in various fields, such as industrial, pharmaceutical, and chemical safety. In this context, Levine *et al.* report on a sensor with boronate ester-functionalized bimane, which responds to the presence of water in organic solvents and water vapor in high humidity environments giving rise to a rapid and sensitive fluorescence quenching (Pramanik et al.). This response occurs both in the bimane solution and on filter papers on which the bimane is adsorbed, resulting in colorimetric and fluorimetric changes. This sensor has notable practical advantages, such as high sensitivity, non-toxicity, and ease of access.

